# Attitudes of young adults from the UK towards organ donation and transplantation

**DOI:** 10.1186/2047-1440-2-9

**Published:** 2013-05-17

**Authors:** Laura Coad, Noel Carter, Jonathan Ling

**Affiliations:** 1 Department of Pharmacy, Health & Wellbeing, University of Sunderland, Sunderland SR1 3SD, UK

**Keywords:** Organ donation, opt-out, Attitudes, Questionnaire

## Abstract

**Background:**

This study examines the attitudes of young British adults towards donating their own organs and those of their family members.

**Methods:**

An opportunity sample of 119 participants (65 female) completed an attitude questionnaire.

**Results:**

Most participants were in favour of donation though substantially fewer had signed up to the organ donation register. A minority of participants was aware of the proposed opt-out system for donation.

**Conclusions:**

The results from this study corroborate and extend previous work in that more participants were prepared to receive an organ than donate one. Knowing someone who had donated an organ was associated with a more positive attitude towards donation. Implications for policy are discussed.

## Background

In 2011 to 2012 the number of patients who died whilst on the UK transplant list was 508 and a further 819 were removed as their health had deteriorated and they would have died shortly afterwards [1]. Historically, the UK has had one of the lowest rates of organ donation in Europe, with only 12.6 people in every million donating organs in 2006, although by 2010 this rate had risen to 27.4 people in every million, close to the European average. This contrasts with Spain, which has one of the highest rates of donation with 42.2 people in every million opting to donate their organs [1]. Although Spain has had a system since 1979 where individuals are assumed to agree to their organs being donated unless they actively opt out, the high rates of donation in Spain have only come about in the past 15 years in response to addressing barriers to organ donation such as ensuring all potential organ donors’ relatives are approached by a specifically trained member of staff [2]. If the UK follows the Spanish example, such as by ensuring families of potential donors are in contact with pro-donation intensive care nurses [3-5], the donation rate could be increased further.

Over the past decade there has been a steady rise in the number of people in the UK who have signed up to the donor register, from 10.2 million in 2002 to 18.7 million registrants by 2012 [1]. Positive though this is, it is much lower than the 90% of the population who claim to support organ donation [6]. Also, in practical terms, if a patient has signed up to the organ register the family’s wishes can and often do override the wishes of the deceased [7]. Little quantitative research has been carried out in the UK investigating attitudes towards organ donation, and the quantitative work conducted outside the UK has predominantly surveyed the attitudes of health professionals [8,9], who tend to have favourable attitudes towards organ donation, but which may not be representative of the general population as medical staff have more insight into the subject of organ donation and transplantation.

As well as examining attitudes towards organ and tissue donation and transplantation and the reasons behind those specific attitudes, one issue of current interest is the attitude of the public towards the proposed opt-out system of organ donation. At present in the UK, in order to donate an organ or tissue after death, an individual must have signed up to the organ donor register - an opt-in system. The opt-out system is where it is presumed that an individual wishes to be a donor unless he or she has opted out by registering their objection to donating their organs. Changing to an opt-out system has been shown to result in an increase in donation [10], as well as increasing the willingness of individuals to donate their organs [11,12]. Such a move, however, would not necessarily have widespread support [13] and it is also possible that reported increases are likely to be also due to other changes such as an increase in transplant coordinators or financial incentives [14]. Furthermore, ethical concerns relating to informed consent also remain [15,16].

Only one quantitative study has been carried out in the UK on the issue of the proposed opt-out system [17]. The study examined the attitudes of the UK’s faith leaders to organ donation because cultural issues could potentially impact on attitudes towards donation. Although the faith leaders were supportive of organ donation, they did not agree with adopting an opt-out system. Christianity and Judaism tend to be more accepting of transplantation generally than other religions [17]. However, in a country that is increasingly secular, whether opinions such as those reported by Randhawa *et al*. [17] are representative of the general population has yet to be determined.

## Methods

### Participants

The 119 (65 female, 54 male) participants had a mean age of 22.7 years (range 18 to 30, SD 3.75). The majority were either employed (52.1%) or in education (37.8%), with the remainder unemployed (6.7%) or working in a voluntary capacity (3.4%). Most participants (46.2%) were educated to college level (equivalent to high school), almost a third was educated to degree level (31.1%), and some also had postgraduate education (9.2%). A minority of participants (13.4%) was educated to secondary level.

Approximately two thirds (65.5%) of participants identified themselves as Christian, with the next highest group those who had no religion or were atheists (23.5%). Participants also identified themselves as Buddhist (4%), Muslim (1%), Sikh (1%) or other (5%). The overwhelming majority identified themselves as being white (93%), with others identifying themselves as Chinese (4%), other Asian (non-Chinese, 1%), mixed race (1%) or African (1%).

The study was performed in accordance with the Declaration of Helsinki, local laws, and other regulations. The study was evaluated and approved by the University of Sunderland Ethics Committee.

### Materials and procedure

Participants completed a questionnaire consisting of 42 questions divided into 5 sections. These were: demographic information; knowledge about organ and tissue donation and transplantation; attitudes towards organ donation; attitudes towards organ transplantation and reasons for attitudes towards organ donation and transplantation. The questionnaire took approximately 10 minutes to complete.

The opportunity sample of young people was recruited from Liverpool city centre by a single researcher (LC). Those taking part in the study did so voluntarily after being fully informed about the research. All data gathered were anonymous and confidential.

## Results

Preliminary analysis of the scale indicated that there were no significant main effects or interactions related to religion, education or employment status, therefore these variables were not analyzed further. The scale had good internal consistency reliability (Cronbach’s alpha = 0.947).

### Knowledge of organ and tissue donation and transplantation

A large majority of participants was familiar with the terms organ donation and organ transplantation, though only slightly over a third had heard of the proposed opt-out system. Few participants had donated or received an organ, but more than a fifth knew someone who had received a donated organ, and almost a tenth knew someone who had donated one (Table 1).

**Table 1:** Knowledge about organ donation

	**Yes**		**No**	
	**n**	**%**	**n**	**%**
Have you heard of the term organ donation?	113	95	6	5
Have you heard of the term organ transplantation?	113	95	6	5
Have you heard of the proposed opt-out system?	43	36.1	76	63.9
Have you ever donated an organ?	2	1.7	117	98.3
Have you ever received an organ for transplantation?	1	.8	118	99.2
Do you know anyone who has donated an organ?	11	9.2	107	89.9
Do you know anyone who has received an organ for transplantation?	25	21	95	79.8

n, number of respondents.

### Attitudes towards organ donation

Attitudes towards donation were generally positive, with nearly two-thirds of participants either agreeing or strongly agreeing with donating an organ for transplantation and almost 90% agreeing or strongly agreeing with receiving one (see Table 2).

**Table 2:** Attitudes towards organ donation and barriers to donation

	**Strongly disagree**	**Disagree**	**Neither agree nor disagree**	**Agree**	**Strongly agree**
What is your general attitude toward donating an organ for transplantation?	5.0 (6)	2.5 (3)	2.5 (34)	33.6 (40)	30.3 (36)
What is your general attitude toward receiving an organ for transplantation?	3.4 (4)	3.4 (4)	15.1 (18)	41.2 (49)	37.0 (44)
My religion does not agree with organ donation or transplantation.	44.5 (53)	16.8 (20)	31.1 (37)	5.9 (7n )	1.7 (2)
I have a cultural belief that my body should be kept intact after death.	45.4 (54)	19.3 (23)	19.3 (23)	10.9 (13)	5.0 (6)
I have a fear that my body will be disfigured if I donate my organs, therefore it makes me less likely to donate.	33.6 (40)	25.2 (30)	22.7 (27)	10.9 (13)	7.6 (9)
I have a fear of surgical procedures	23.5 (28)	21.8 (26)	26.9 (32)	17.6 (21)	10.1 (12)
I distrust the NHS	22.7 (27)	34.5 (41)	26.9 (32)	12.6 (15)	3.4 (4)
My family does not agree with organ donation	33.6 (40)	25.2 (30)	30.3 (36)	5.0 (6)	5.9 (7)

Scores are presented as percentages (frequencies) from a sample of 119 respondents. NHS, National Health Service.

### Relationship between knowledge and attitudes towards organ donation and transplantation

One positive finding is that the overwhelming majority of participants had heard of the terms organ donation and organ transplantation. However, as so few participants (6/119) had not heard of the term, any statistical comparisons made would be underpowered. Participants who had heard of the proposed opt-out system were more likely to have a positive attitude towards organ donation (mean = 4.12) than those who had not (mean 3.64, *t* = 2.83, df = 117, *P* = 0.019), and there was a trend towards them being more positive towards receiving a donated organ (mean 4.42 and 3.84, respectively, *t* = 3.20, df = 117, *P* = 0.065).

### Knowledge of somebody that has donated or received an organ

Most participants did not know either an organ donor (90.8%) or a recipient (79%), although whether someone knew a donor or a recipient had no influence on attitudes towards their own organs being donated, or whether they agreed with the opt-out system.. Interestingly, there was a trend towards a difference in attitudes towards donating the organs of their family members (*t* = 1.84, df = 117, *P* = 0.068), with participants who had themselves, or knew someone who had received or donated an organ, more likely to agree with donating the organs of family members than those who did not (means 4.03 and 3.63, respectively).

## Discussion

One of the aims of this study was to determine the proportion of participants who reported that they would donate their own organs. We found that almost two thirds of participants agreed with donating their own organs, compared to just over a quarter who neither agreed nor disagreed; few participants disagreed with donating their own organs. The proportion of those in favour of donating their own organs is much lower than the 90% who claim to support organ donation reported by the NHS Blood and Transplant (NHSBT) [6]. The proportion of participants who stated that they would agree to donating their own organs is 36.9% lower than those signed up to the NHS Organ Donor Register (27%) at the time the questionnaire was carried out. Since then, the number of adults on the organ register has risen to approximately 38% of the adult population (based on an estimated 49.1 million adults in the UK in 2012). There could be several reasons for this: perceived difficulty of signing up to the Organ Donor Register, lack of information about what is required to join the register or just that signing up is not a priority. Many of these issues could be overcome by introducing either the opt-out (presumed consent) system of organ donation or the system of automatic consent registration. It is clear that the positive data from this survey reflect an increased awareness and willingness to donate in the UK.

The present study also aimed to discover the proportion of participants who said they would receive an organ for transplantation. We found that more than three quarters of our participants reported that they would agree to be a recipient of an organ for transplantation. This replicates findings from previous work [18] that indicate that the willingness to receive an organ for transplantation is usually higher than the willingness to donate one. In the present study 63.9% of participants were willing to donate their organs compared to 78.2% of participants who were willing to receive one.

A further aim of this study was to find out the extent to which participants agreed with donating their family members’ organs. This is an important issue, given that in the UK the wishes of relatives can override those of an individual who has placed their name on the organ donation register. While a majority stated that they were in favour of donating family members’ organs, this was lower than the level for donating their own organs. This supports other work, which has found that in almost half of cases relatives will refuse to donate organs even when the potential donor was on the donor register [19]. More work is needed to understand why relatives refuse to donate the organs of potential donors, although recent research has indicated that relatives are more likely to support the wishes of the deceased registered donor when they have previously discussed their wishes with them [20]. Where, hitherto the focus has been almost entirely on encouraging more people to join the register, perhaps now campaigns should encourage registrants to discuss their wishes with their relatives. At a time when there are far more potential recipients than donated organs available, improving the numbers of organs made available through prompting people to have discussions with their relatives about their wishes would be an important step to achieve this.

The proposed opt-out (presumed consent) system of organ donation was supported by a slight majority of participants, with a quarter expressing no preference, and a quarter disagreeing or strongly disagreeing. A recent systematic review showed that prior to 2000 within the UK, most people opposed an opt-out or presumed consent system. A systematic review of four studies conducted since 2000 showed that on average 60% of people surveyed are in favour of donation by presumed consent [21]. There was no significant difference between the sexes in attitudes towards organ donation. However there was a trend towards female respondents being more likely to agree to donate relatives’ organs.

There was no significant difference between religious and non-religious participants on any of the questions, nor was there an effect of either education or employment status.

### Limitations

The lack of participants from different socio-demographic backgrounds was a limitation of this study. In particular, the lack of substantial numbers of people from non-Christian faiths makes comparisons with the data obtained by Randhawa *et al*. [17] difficult. However, this mirrors observations nationally that those from higher socio-economic groups tend to register as donors [1].

In summary, the results from this study corroborate the NHSBT [6] findings that 90% of the population are in favour of organ donation. There was, however, still a considerable difference in the proportion of people that reported agreeing with donating their own organs and those that are actually signed up to the NHS Organ Donor Register. This gap could be reduced by introducing either the opt-out (presumed consent) system of organ donation or the system of automatic consent registration [15]. Similar to the Spanish experience, alternative approaches to increasing the numbers of individuals on the organ donation register in the UK appear to have been successful. A major success in the UK has been to link registration on the register to the application of a new driving licence: in 2012, 57% of all new registrants came via the Driver and Vehicle Licensing Agency (DVLA) [1]. The NHS has also embarked on a major educational and marketing campaign for organ donation and, anecdotally, there appears to be an increase in positive donation stories in the media. This has not been studied in a European context, but a recent study in Venezuela demonstrated that positive media interest can have a significant effect on organ donation rates [22]. This offers a potentially interesting area for further study.

A majority of participants agreed with the opt-out system; although we do not provide conclusive evidence this was likely to be related to their knowledge of people who had donated or received a donated organ. This implies that increasing awareness of organ donation through education could have a positive impact on donation rates, even if presumed consent is not pursued in the UK. A potential issue for the future may arise through the increasing use of organs retrieved where the donors have died from cardiac arrest rather than brainstem death, so-called donors by cardiac death (DCD). This is the fastest growing source of donor organs in the UK [1]. A recent systematic review highlights concerns in both the medical profession and the general population about the use of such organs [23].

## Competing interests

The authors declare that they have no competing interests.

## Authors’ contributions

LC conceived the study and participated in its design and coordination and collected the data. JL participated in the design of the study and helped draft the manuscript. NC helped to draft the manuscript. All authors read and approved the final manuscript.
